# A novel causative LMNA variant in acrogeria syndrome and literature review of genotype–phenotype correlations

**DOI:** 10.1016/j.gendis.2026.102283

**Published:** 2026-06-09

**Authors:** Haisheng Huang, Yingying Miao, Yumeng Wang, Rui Lei, Mo Zhang, Cheng Zhang, Xianwei Cao, Ming Li, Yunqing Ren

**Affiliations:** a Department of Dermatology, The Children's Hospital of Fudan University, Shanghai 201102, China; b Department of Dermatology, Hangzhou Children's Hospital, Hangzhou, Zhejiang 310014, China; c Department of Dermatology, The First Affiliated Hospital, Jiangxi Medical College, Nanchang University, Nanchang, Jiangxi 330006, China; d Department of Dermatology, Children's Hospital, Zhejiang University School of Medicine, National Clinical Research Center for Child Health, Hangzhou, Zhejiang 310006, China

Acrogeria (OMIM #201200) is an extremely rare genetic dermatosis first reported by Gottron in 1940. The clinical manifestations include cutaneous atrophy with mottled pigmentation and subcutaneous tissue atrophy, mainly affecting acral regions (hands and feet), with pronounced aging that far exceeds the patient's chronological age.^1^ Acrogeria arises from pathogenic variants in either *LMNA* or *COL3A1*, predominantly following an autosomal dominant inheritance pattern. However, an autosomal recessive inheritance pattern has been observed in rare familial cases. The *LMNA* gene encodes type A nuclear lamellar proteins through selective splicing of Lamin A and C (Lamin A/C), which play crucial roles in maintaining nuclear structure and function and in regulating gene transcription.^2^ Pathogenic *LMNA* variants underlie a spectrum of clinically heterogeneous genetic disorders collectively classified as laminopathies (OMIM #150330). While sharing clinical features with other laminopathies (*e.g.*, Hutchinson–Gilford progeria syndrome, mandibuloacral dysplasia, and restrictive dermopathy), acrogeria is pathognomonically characterized by predominant acral involvement.^1^ Additionally, vascular Ehlers-Danlos syndrome (OMIM #130050), resulting from *COL3A1* mutations, demonstrates phenotypic overlap with acrogeria. The difference is that acrogeria does not present characteristic features such as cigarette-paper scars and intestinal catastrophes.^3^

A 32-year-old male proband presented at the dermatology clinic with mottled cutaneous pigmentation. The proband had developed progressively expanding pigmented macules throughout his body since the age of seven, accompanied by emaciation and short stature. Currently, he had a height of 166.2 cm and a weight of 42.5 kg, with a body mass index (BMI) of 15.39 kg/m^2^. According to the WHO classification criteria for adult nutritional status, this BMI falls into the category of severe thinness (BMI < 16.0 kg/m^2^). Routine physical examination disclosed no additional significant abnormalities. The proband's physical examination revealed distinctive features, including a prominent nasal bridge, generalized hyperpigmented macules with predominant distribution over limb joints, and markedly aged-appearing hands characterized by atrophic dermis, prominent venous patterning, reduced subcutaneous adipose tissue, and mottled pigmentation (Fig. 1A). The proband's 6-year-old daughter initially developed hyperpigmented macules on the dorsum of her hands two years ago, whose physical examination revealed scattered hyperpigmented patches with similar characteristics to those of the proband, distributed over the hands and feet, periarticular regions of the extremities, buttocks, and trunk (Fig. 1B). The proband reported that his mother displayed similar cutaneous manifestations, along with an asthenic build and short stature, and exhibited accelerated aging after 40 years of age relative to peers. No other family members, including the patient's two sisters, exhibited comparable phenotypic features (Fig. 1C).

**Figure 1:**
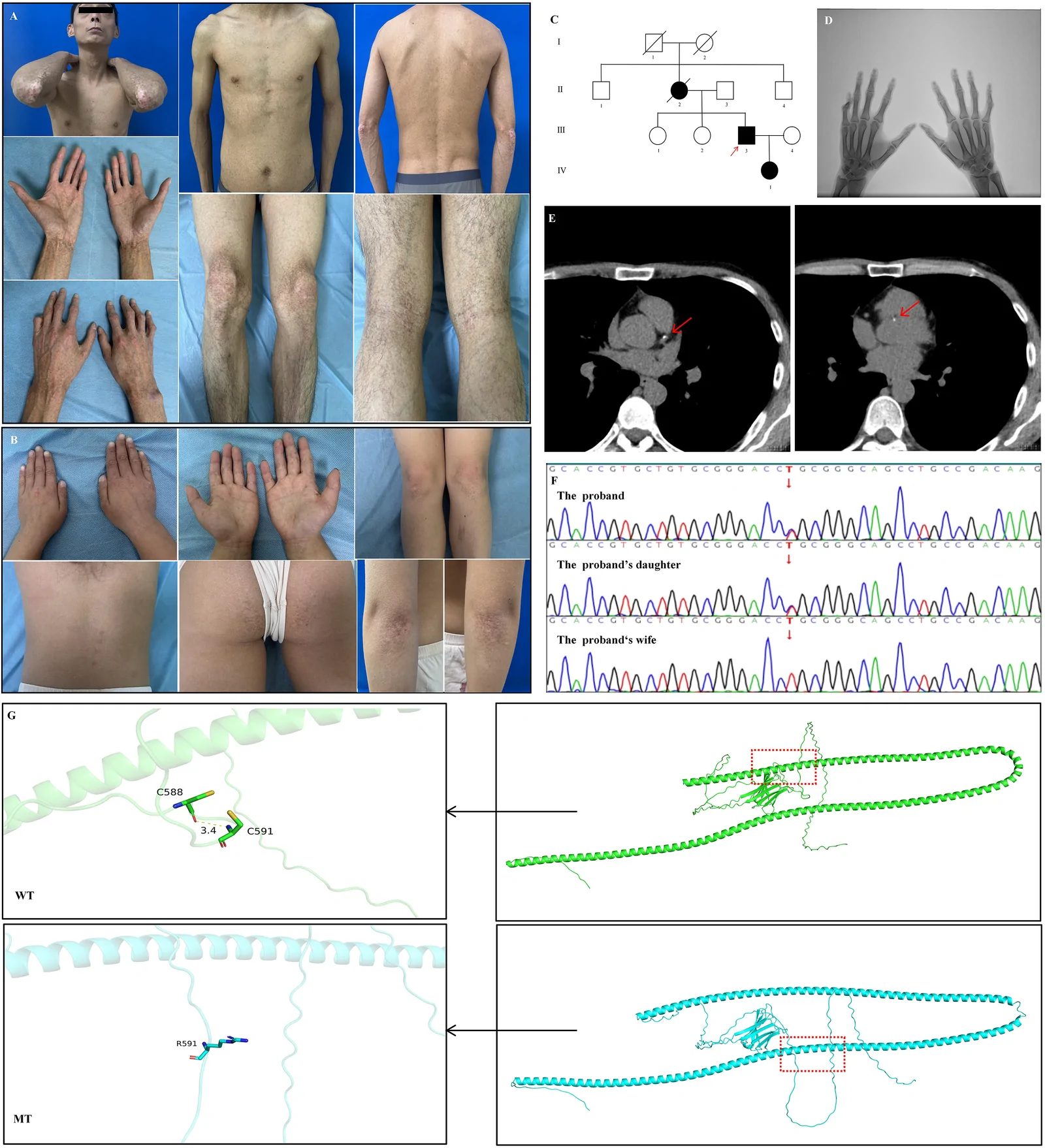
A novel causative *LMNA* variant in a family with acrogeria syndrome. **(A)** Clinical features of the proband: prominent nasal bridge; generalized hyperpigmented macules with predominant distribution over limb joints; markedly aged-appearing hands characterized by atrophic dermis, prominent venous patterning, and reduced subcutaneous adipose tissue accompanied by mottled pigmentation. **(B)** Clinical features of the proband's daughter: scattered hyperpigmented patches distributed over the hands, feet, periarticular regions of the extremities, buttocks, and trunk. **(C)** Pedigree of the family with Acrogeria. The arrow indicates the proband. The slash indicates deceased. **(D)** The hand X-ray of the proband. **(E)** The proband's coronary computed tomographic angiography showing calcification (red arrows) in the left anterior descending artery (LAD, left) and the left ventricular outflow tract (LVOT, right). **(F)** The proband and his daughter have a missense pathogenic variant c.1771T>C in the exon 11 of *LMNA.***(G)** Three-dimensional structure of *LMNA*. WT, wild-type; MT, mutant type.

The proband's hand X-ray examination revealed reduced bone density at the metaphyses of both hands, along with suboptimal alignment of the fifth proximal interphalangeal joints (Fig. 1D). Coronary computed tomographic angiography revealed a calcified plaque in the proximal segment of the left anterior descending (LAD) artery with mild luminal stenosis, myocardial bridging in the mid-segment of the LAD, CAD-RADS category 1, and calcification of the left ventricular outflow tract (LVOT) wall (Fig. 1E). The coronary artery calcium score was 1. Echocardiography revealed mild mitral, tricuspid, and aortic regurgitation. Blood biochemical tests revealed that non-esterified fatty acids (NEFA) level was elevated at 997 μmol/L (reference range: 172–586 μmol/L), while the electrocardiogram and other routine blood tests showed no significant abnormalities. The proband's daughter showed no significant abnormalities on electrocardiography, hand X-ray, and routine laboratory blood tests.

The investigation was approved by the Human Medical and Ethical Committee of the Children's Hospital of Fudan University, and all subjects provided informed consent. After obtaining the collected blood samples of the proband, whole exome sequencing was performed. A heterozygous pathogenic variant in exon 11 of the *LMNA* gene (c.1771T>C; p.C591R) was detected, which was not present in the gnomAD database. *In silico* analysis using PolyPhen-2 predicted a possibly damaging effect with a score of 0.917. Based on the ACMG guidelines, this variant was classified as a variant of uncertain significance. Sanger sequencing was employed to validate the proband's family and 100 unrelated healthy controls. The results indicated that the proband and his daughter carried this pathogenic variant, while no base substitutions were found in the unaffected individuals or control group (Fig. 1F). We further screened the variant in an ethnically matched cohort of over 20,000 healthy individuals from the database of Shanghai WeHealth Biomedical Technology Co., Ltd. (Shanghai, China), and it was not detected. Unfortunately, since the affected proband's mother had passed away, her DNA was unavailable for analysis.

The identified pathogenic variant (reference sequence: NM_170707.4) in exon 11 is a missense pathogenic variant, and there are no relevant reports currently. This site is located within a C-terminal specific region (residues 567–664) unique to Lamin A, which is absent in Lamin C due to its premature termination at position 566. The monomer_casp14 model from AlphaFold v2.3.2 was used to predict the three-dimensional structure of LMNA, which showed that the primary difference between wild-type and mutant proteins lay in the substitution of cysteine (C) to arginine (R) at position 591, located within the C-terminal disordered region. Structural comparison revealed that the main spatial structure remained basically unchanged between the two variants. The primary structural distinction resided in the disruption of the C591–C588 hydrogen bond in the mutant variant, an interaction that was maintained in the WT protein (Fig. 1G).

Acrogeria is characterized primarily by premature aging of distal extremities, typically without cardiac involvement. The proband in this report had a heterozygous pathogenic variant of the LMNA gene (c.1771T>C; p.C591R), and his cardiac function showed no significant organic lesions. However, according to the recent Japanese population study by Takagi et al, a coronary artery calcium score of 1 in a 35–44-year-old male already exceeds the 90th percentile of his healthy peers, suggesting that his vascular biological age is significantly advanced and his cardiac functional reserve is markedly poorer than that of individuals of the same age.^4^ The proband described that his mother had a history of poor cardiac function, classified as NYHA Ⅲ. At age 55, she experienced a sudden onset of symptoms at home without warning and passed away during transport to the hospital. The exact cause of death remained unclear, but it was presumed to be cardiac-related. We summarized the previously reported cases with confirmed genetic mutations to investigate potential genotype–phenotype correlations (Table S1). A total of four genetically confirmed cases of Acrogeria have been previously reported, with ages at diagnosis predominantly in the 30s and 40s; all patients exhibited premature aging-like hand manifestations and various forms of skin atrophy. Intriguingly, among them was a 36-year-old male patient carrying the same mutation site (c.1771T>A; p.C591S) as our proband.^5^ His father—who shared similar cutaneous manifestations but passed away without genetic confirmation—had undergone mitral and aortic valve replacement. Regrettably, due to the lack of specific case information and genetic diagnosis of his father and the proband's mother, we were unable to further elucidate the genotype–phenotype correlation.

Acrogeria, an exceptionally rare genetic dermatosis, has been reported in only approximately 50 cases worldwide to date, with the majority documented before 2000 and merely five cases reported in the past two decades. We report a familial case with classic dermatological manifestations accompanied by suspected cardiac abnormalities. The lack of definitive genetic diagnoses in most reported cases has significantly hampered investigations into correlations between genotype and cardiac abnormality. However, we contend that regular cardiac and coronary evaluations remain imperative for acrogeria patients, particularly those harboring pathogenic *LMNA* variants.

In conclusion, we report this unreported pathogenic variant c.1771T>C (p.C591R) with potential cardiac abnormalities to expand the database of *LMNA* gene pathogenic variants in Acrogeria, which is helpful for further studying the relationship between pathogenic variations of *LMNA* and different phenotypes.

## CRediT authorship contribution statement

**Haisheng Huang:** Writing – review & editing, Writing – original draft. **Yingying Miao:** Writing – original draft. **Yumeng Wang:** Writing – review & editing. **Rui Lei:** Writing – review & editing. **Mo Zhang:** Writing – review & editing. **Cheng Zhang:** Supervision. **Xianwei Cao:** Resources. **Ming Li:** Resources, Formal analysis, Conceptualization. **Yunqing Ren:** Writing – review & editing, Conceptualization.

## Ethics declaration

This study was approved by the ethics committees of Children's Hospital of Fudan University (No. (2023) 31). Written informed consent for publication of clinical details and images was obtained from the patients' family.

## Data availability

All data described in this manuscript are available.

## Funding

This work was supported by grants from the 10.13039/501100001809National Natural Science Foundation of China (No. 82073422, 82273504).

## Conflict of interests

The authors declared no conflict of interests.
