# Supplementary material for: A novel causative LMNA variant in acrogeria syndrome and literature review of genotype–phenotype correlations
Source: Genes Dis. 2026 Jun 9;14(1):102283. doi: 10.1016/j.gendis.2026.102283 (PMC13542494; doi:10.1016/j.gendis.2026.102283)
Supplement: Supplementary file 1 [file mmc1.docx]

**materials and methods**

1. **Whole Exome Sequencing (WES)**: Genomic DNA was extracted from patient EDTA-anticoagulated blood samples, and the quality and concentration of the extracted DNA were assessed. The genomic DNA was then fragmented, end-repaired, adapter-ligated, and PCR-amplified to construct a sequencing library. Whole-exome enrichment was performed through hybrid capture using the IDT xGen® Exome Research Panel v2.0 probes to enrich target region DNA fragments. Sequencing was carried out on the MGI DNBSEQ-T7 platform. The sequencing reads were aligned to the Ensemble reference genome GRCh37/hg19 using Burrows-Wheeler Aligner (BWA) software, followed by SNP and Indel analysis with GATK. Detected SNPs and Indels were subsequently filtered and screened based on sequencing depth and mutation quality to obtain high-confidence variants. Finally, these high-quality variants were annotated and assessed for variant deleteriousness and prediction using variant annotation software.
2. **Sanger sequencing:** Primers were designed corresponding to the variant sites. Amplification was performed using PCR. The PCR reaction conditions were as follows: initial denaturation at 95 °C for 5 min; 30 cycles of denaturation at 95 °C for 30 s, annealing at 60 °C for 30 s, and extension at 72 °C for 30 s; followed by a final extension at 72 °C for 10 min. The PCR system volume was 20 μL. The amplified PCR products were validated by 1% agarose gel electrophoresis and subjected to Sanger sequencing using the ABI 3730XL sequencer (Applied Biosystems, USA) to validate the candidate variant sites and determine the origin of the variants. DNASTAR software was used for sequence analysis and alignment.
3. **Protein three-dimensional structure prediction:** The amino acid sequence of LMNA was downloaded from the UniProt database, and the amino acid sequence of the mutant protein was derived based on the genetic variant information. The sequences were compiled in FASTA format. The structures of both the wild-type and mutant proteins were predicted using AlphaFold Server, and the prediction model with the highest confidence (pLDDT) was analyzed using PyMOL v3.1.3.
