# Supplementary material for: A novel causative LMNA variant in acrogeria syndrome and literature review of genotype–phenotype correlations
Source: Genes Dis. 2026 Jun 9;14(1):102283. doi: 10.1016/j.gendis.2026.102283 (PMC13542494; doi:10.1016/j.gendis.2026.102283)
Supplement: Supplementary file 2 [file mmc2.docx]

**Table S1.**

|  | Our case | Valayer S et al.2024 | Maiese A et al.2019 | Hadj-Rabia S et al.2014 | Jansen T et al.2000 |
| --- | --- | --- | --- | --- | --- |
| **Age of initial diagnosis** | 32 | 32 | 29 | 36 | 43 |
| **Sex** | M | F | M | M | M |
| **Family history** | Proband's daughter: Affected  Proband's mother: Suspected | _ | _ | Proband's father: Suspected | **_** |
| **Key Clinical phenotype** | hands aging；  prominent nasal bridge；  general mottled pigmentation | beaked nose;  an ‘owl eyed’ appearance;  tan atrophic skin, no dyspigmentation | brachydactyly with acrosclerosis;  palmoplantar pitted keratoderma;  moderate ichthyosis, predominantly on lower limbs | hands aging；  thin nose and translucent skin;  localized mottled pigmentation | sharp and slim nose;  hands aging with mottled pigmentation |
| **Cardiac abnormalities** | Proband's mother: NYHA class Ⅲ | _ | _ | Proband's father: mitral and aortic valve replacement | **_** |
| **Pathogenic gene** | *LMNA*;p.Cys591Arg | *LMNA*;p.Ala375Val | *COL3A1* | *LMNA*;p.Cys591Ser | *COL3A1*;p.Gly1006Arg |
